# It's not just a phase: Investigating text simplification in a second language from a process and product perspective

**DOI:** 10.3389/frai.2022.983008

**Published:** 2022-09-12

**Authors:** Alessandra Rossetti, Luuk Van Waes

**Affiliations:** Department of Management, Faculty of Business and Economics, University of Antwerp, Antwerp, Belgium

**Keywords:** text simplification training, second language, writing phases, text readability, keystroke logging, automated text analysis

## Abstract

Text simplification involves making texts easier to understand, usually for lay readers. Simplifying texts is a complex task, especially when conducted in a second language. The readability of the produced texts and the way in which authors manage the different phases of the text simplification process are influenced by their writing expertise and by their language proficiency. Training on audience awareness can be beneficial for writers, but most research so far has devoted attention to first-language writers who simplify their own texts. Therefore, this study investigated the impact of text simplification training on second-language writers (university students) who simplify already existing texts. Specifically, after identifying a first and a second phase in the text simplification process (namely, two distinct series of writing dynamics), we analyzed the impact of our training on pausing and revision behavior across phases, as well as levels of readability achieved by the students. Additionally, we examined correlations between pausing behavior and readability by using keystroke logging data and automated text analysis. We found that phases of text simplification differ along multiple dimensions, even though our training did not seem to influence pausing and revision dynamics. Our training led to texts with fewer and shorter words, and with syntactically simpler sentences. The correlation analysis showed that longer and more frequent pauses at specific text locations were linked with increased readability in the same or adjacent text locations. We conclude the paper by discussing theoretical, methodological, and pedagogical implications, alongside limitations and areas for future research.

## Introduction

Text simplification can be defined as the modification of written language in order to make it more understandable (Crossley et al., 2012). As such, text simplification is a complex and cognitively demanding activity which—in order to be successful—requires awareness about the audience and knowledge about the types of edits that will make a text easier to read (Schriver, 2012). Levels of writing expertise and (second) language proficiency can influence the process by which text simplification is produced, as well as the quality of its products (e.g., the readability of the final texts) (Hayes et al., 1987; Hayes, 2004; Barkaoui, 2016). For instance, experienced writers are able to simultaneously take into account the reader, the text, and the intentions of the author (Kellogg, 2008). Some types of training have been found to assist students in adapting their texts to the comprehension needs of the readers (Schriver, 1992).

In this study, we used keystroke logging and automated text analysis in order to expand current knowledge about the process and the product of text simplification in a second language (L2). Specifically, we examined the extent to which text simplification training influences the writing dynamics of L2 students (process perspective) and the readability of their texts (product perspective).

This paper is structured as follows. The Section Literature review reviews previous research about the writing process, writing quality, and the impact of training. Furthermore, the Section Literature review highlights the research gaps and the research questions that we addressed in our study. The Section Materials and methods reports on the methodology adopted—participants, design, procedure, texts, and training—and on the data analysis procedure. The Section Results presents the results, which are then discussed in the Section Discussion, along with their theoretical, methodological, and pedagogical implications. We conclude the paper by outlining limitations and avenues of future research.

## Literature review

### Writing expertise and the writing process

Writing expertise—and the training that can foster it—has received substantial attention, especially from a cognitive perspective (Kellogg and Whiteford, 2012). Already in the 1980s, researchers had observed that more experienced writers tend to regularly revise their texts and their writing goals, in a recursive, non-linear fashion (Sommers, 1980; Flower and Hayes, 1981). In other words, far from regarding revision as a separate phase at the end of linear text production, skilled writers revise and rewrite as they write (Sommers, 1980). Their goal is to ensure that the text matches the author's intention (knowledge-transforming) or—at a more advanced stage of writing expertise—that the text matches the needs and preferences of the intended readers (knowledge-crafting) (Bereiter and Scardamalia, 1987; Kellogg, 2008; Schriver, 2012).

The increased use of the computer and word processing software has provided a new perspective for the investigation of writing processes (Van Waes and Schellens, 2003). In particular, the development of keystroke logging tools—such an Inputlog, Scriptlog, or Translog—has allowed researchers to collect fine-grained data on keys pressed, pauses (e.g., duration, number, distribution), fluency, and revisions (e.g., number, type, and location), in turn shedding light on the cognitive processes that underlie writing (Leijten and Van Waes, 2013). For instance, Lindgren et al. (2011) used the keystroke logging data provided by Inputlog to analyze and compare the processes by which writers with different years of expertise adapt texts to the reader. A key component of their analysis relied on the Inputlog process graphs, which are visual representations of pausing and revision behavior, source use, and cursor movements throughout the writing task (Vandermeulen et al., 2020). Lindgren et al. (2011) observed that novice writers and experienced writers had different revision patterns, with the latter revising the initial draft from top to bottom multiple times in a cyclical and non-linear way and devoting each round of revision to a specific aspect of the text.

### The (non-) linearity of the writing process

If we consider linear writing as text production at the point of utterance (i.e. the point at which new text is being produced)—with no (major) back movements to previous parts of the document—then revisions represent a break in the linearity of the writing process (Buschenhenke et al., under review; Perrin and Wildi, 2008). Researchers have identified different profiles based on writing linearity by taking into consideration the timing, location, and scope of revisions. Eklundh (1994), for instance, reports on an article by Williamson and Pence (1989), who outline the profiles of the linear reviser, the intermittent reviser, and the recursive reviser. The *linear reviser* first composes a full draft of their manuscript—making only local edits at the word and sentence levels—and then scrolls back to the start of their draft to make more substantial changes throughout the text. The *intermittent reviser* alternates periods of linear text production with back movements to the preceding paragraphs or chunks, where revisions are mainly carried out at the phrase or clause level. The *recursive reviser* displays shorter periods of uninterrupted, linear text production as they regularly revise different text elements—from words to sentences—especially close to the point of utterance (Williamson and Pence, 1989). Van Waes and Schellens (2003), on the other hand, identify five profiles, including *fragmentary stage I writers* and *stage II writers*. The former carry out most of their revisions while producing a first draft, show a high degree of recursion, and make few revisions after the first draft is written. *Stage II writers*, on the other hand, make the majority of revisions after the first draft is completed, show a low degree of recursion, and carry out many revisions above the word level. In a similar study, Levy and Ransdell (1995) identified processing profiles (or *writing signatures*) as they observed consistent and distinctive patterns in the recursive shifts between the sub-processes of planning, generating, and revising texts.

Recursive, non-linear movements during writing can be motivated by the need to revise the text-produced-so-far, to read the previous text, or to consult external sources. Perrin and Wildi (2008) make a connection between linearity and stages of writing, as they argue that most writing processes do not follow a linear pattern since the three main cognitive sub-processes of writing—usually identified as planning, formulating, and reviewing content—intertwine and are each characterized by specific, dominant dynamics reflected in the cursor, mouse, and keyboard activity. For example, while re-reading previous content for new ideas usually takes place during the phase of formulation, re-reading of a whole text to check its sense and structure usually takes place during the final revision phase. This type of whole-text re-reading is usually carried out by expert writers who are able to build a global representation of the text, while novices tend to be mainly concerned with low-level revisions at the word and sentence level (Schaeffer et al., 2019). Perrin and Wildi (2008) also specify that the same practices can be adopted throughout the entire writing process, even though with a different frequency. Interestingly, at the end of their paper, Perrin and Wildi (2008) suggest investigating the impact of training on writing phases, which is the focus of our paper (Section Research gaps and research aims).

### The temporal organization and dynamics of the writing process

Even though Van den Bergh and Rijlaarsdam (1996) demonstrated the importance of a more time-based approach to the analysis of writing, the temporal organization of writing processes has only received limited attention. Such temporal organization seems to depend on the writers' profiles. In other words, depending on their profiles, writers might distribute cognitive activities differently throughout a writing task. Research by Xu and Xia (2021) is a good example of a keystroke-logging study that takes into account this temporal organization of the writing process. In their article, the authors divided the L2 writing process into the three traditional sequential phases of prewriting/planning, formulation, and revising/reviewing. Their phase-based analysis revealed that novice L2 writers gave more prominence to formulation, thus limiting the other phases, while more proficient writers better balanced their phase management.

In addition to the temporal organization of writing, scholars have focused on revision dynamics and on the (non-) linearity associated with them, but they differed in their operationalizations and measurements of (non-) linearity (Buschenhenke et al., under review). While some researchers have used approaches requiring manual, content-related identification or annotation of non-linear events as recorded by keystroke logging tools (e.g., any scrolling, deletions, or revisions preceding the production of new text at different levels) (Baaijen and Galbraith, 2018), other researchers have focused on specific types of revisions (such as insertions of large text passages away from the point of utterance) and have relied on S-notation, namely a computer-based method that numbers insertions and deletions according to the order in which they were carried out by the writer (Eklundh, 1994). More recently, Buschenhenke et al. (under review) have adopted a broader view of non-linearity that includes all movements (either with cursor or with mouse) located away from the point of utterance.

The way in which researchers have operationalized phases, stages, or episodes in their studies is also quite different. For instance, a study by Van den Bergh and Rijlaarsdam (2001) divided the writing process into the two main cognitive activities of *task representation* and *writing*, based on data from think-aloud protocols. Xu and Xia (2021), on the other hand, relied on the dynamics observable through keystroke logging data. Similar to our study, the authors used the Inputlog process graphs, and specifically the cursor position being moved to the start of the text, in order to identify the revision phase of the writing process (Xu and Xia, 2021).

However, the most common approach so far—especially in studies relying on keystroke logging—has been to divide the writing process in function of the total task duration. A study by De Larios et al. (2006) divided the writing process mathematically into three equal temporal segments, while a study by Van Waes and Leijten (2015) comparing fluency in first language (L1) and L2 adopted a more fine-grained approach by dividing the process into ten equal time intervals [see also (Leijten et al., 2019b), in which five time intervals were used]. So, we observe a large variety in practices. In this study, we did not use a mathematical, interval-based division of the writing process, but we rather opted for a division of the process content wise, by relying on the process dynamics observable in the process graphs generated by Inputlog (see section Criteria for identifying writing phases).

### Text quality and writing expertise

In addition to (or as a result of) having a more recursive, non-linear writing process, more experienced and professional writers also produce texts of higher quality, with greater lexical sophistication, syntactic complexity, and global cohesion, both in their L1 and L2 (Crossley, 2020). When it comes to adapting texts to the literacy level of the reader, research has shown that the texts written by experienced and linguistically proficient writers tend to be more coherent and in tune with the needs and preferences of the different stakeholders (Alamargot et al., 2010; Schriver, 2012). These achievements in terms of reader-orientedness are linked with the types of revisions that experts apply to their texts, as they make edits at all text levels (from sentences to paragraphs) in order to build or shape arguments and persuade their imagined reader (Sommers, 1980). For instance, complex revisions (such as clarification of meaning) seem to have a positive relation with writing quality, defined as flow of the prose, quality of the arguments, and insight (Cho and MacArthur, 2010). In contrast, novice writers' revisions tend to focus on surface elements and not to alter meaning (Faigley and Witte, 1981). Interestingly, Faigley and Witte (1981) observed that the revisions of advanced university students share similarities with those of both more experienced and less experienced writers, in line with what we also observed in a previous case study [Rossetti and Van Waes, under review (a)]. This observation is particularly relevant considering the cohort of participants in our study (Section Participants). Interestingly, this state of semi-expertise also emerged in a study conducted by Myhill and Jones (2007) with secondary-school students, who showed concern with high-level issues of coherence and general text improvement.

There have been empirical investigations of how differences in the organization of the writing process influence text quality (Van den Bergh and Rijlaarsdam, 2001; Alamargot et al., 2010; Choi and Deane, 2021). For instance, focusing on L2 writing, Xu and Ding (2014) found that more skilled L2 writers paused longer during the prewriting stage, and that longer pauses at the pre-writing stage were correlated with higher text quality in terms of organization, content, and language. In a follow-up study, Xu and Qi (2017) used the keystroke logging tool Inputlog to investigate how proficiency in L2 writing influences pausing behavior and, in turn, text quality. Their results showed that global pausing patterns were similar for more and less proficient writers. However, when dividing the process into intervals, they found that more skilled writers paused more at the initial stage in order to make global plans for their texts. In turn, this behavior had a positive influence on the quality of their texts (Xu and Qi, 2017). In a different study, Beauvais et al. (2011) investigated the relationship between university students' management of the writing process and the resulting text quality. Their results showed that time devoted to planning correlated with text quality for argumentative (but not narrative) texts, and that the lowest percentage of task time was devoted to revising, regardless of the task.

### Readability and text quality

The assessment of text quality (including text readability) is becoming increasingly automatized. Recent developments in Artificial Intelligence and Natural Language Processing have made it possible to develop tools that automatically score texts and, in turn, provide researchers, teachers, and students with feedback on multiple linguistic and rhetorical features (Crossley et al., 2013; Shermis and Burstein, 2013; Crossley, 2020). The majority of these tools include an automatic analysis of text cohesion (i.e. the degree to which ideas in a text overlap and are linked with each other) (McNamara et al., 2014). Global text cohesion is strongly correlated with human evaluations of overall text quality (Crossley and McNamara, 2010, 2011) and has been found to influence reading comprehension (Ozuru et al., 2009). An example of these automatic text scoring tools is TAACO (Tool for the Automatic Analysis of Cohesion), which incorporates around 150 indices related to text cohesion, such as connectives, lexical overlap, and semantic overlap (Crossley et al., 2016). In their study about the predictive validity of the tool, Crossley et al. (2016) found that global—but not local—indices of cohesion calculated at the paragraph level (e.g., overlap of nouns, verbs, and adverbs across paragraphs) correlated with expert human evaluations of text quality.

Coh-Metrix—which we adopted in our study (section Readability data)—is another example of a tool used for the automatic analysis of text readability. This computational and theoretically grounded tool assesses the readability of texts along multiple dimensions (McNamara et al., 2014), informed by theoretical models of reading comprehension according to which, for reading comprehension to succeed, multiple levels of text processing are necessary, from phonology and morphology, to words decoding, sentence interpretation, and the building of inferences by means of background knowledge (Graesser et al., 2014). Currently, Coh-Metrix provides around 300 indices of text readability mainly related to words, syntax, and cohesion (Graesser et al., 2011). For instance, the tool measures referential cohesion (i.e. the extent to which nouns, pronouns, or noun phrases refer to other elements of the text) by measuring co-reference, lexical diversity, and conceptual overlap among sentences (Graesser et al., 2011). Coh-Metrix was developed to address the shortcomings of traditional readability formulas, which evaluated text readability by calculating a limited number of text features (mainly word length and sentence length) (Crossley et al., 2011). However, the current version of Coh-Metrix still provides results of two readability formulas—the Flesch-Kincaid Grade Level and the Flesch Reading Ease—for comparison purposes.

In order to automatically score texts along multiple dimensions, Coh-Metrix relies on different components, such as lexicons, syntactic parsers, and latent semantic analysis (McNamara et al., 2010). Coh-Metrix has been used for the readability analysis of texts written both in L1 and L2 (Baba and Nitta, 2010). With regard to L2 writing, it is worth mentioning the study by Guo et al. (2013), who applied Coh-Metrix to the analysis of both independent and source-based essays (namely essays that require the integration of content from reading or listening resources). Their results on source-based essays showed that writing quality was determined by a number of factors, including text length, lexical sophistication, and cohesion (semantic similarity).

### Training and second-language writing

Learning to simplify texts as expert writers do requires sustained and dedicated practice (Kellogg, 2008), as well as the ability to adopt the perspective of the reader (López et al., 2021). Researchers have investigated the extent to which different types of training help writers to achieve their communicative goals. Training that involves exposing students to the reactions of readers has been found to be beneficial for audience awareness and, in turn, for the anticipation of readers' needs (Schriver, 1992). Rijlaarsdam et al. (2009) have expanded this discussion with suggestions on how to integrate reader observation in the classroom, so as to foster audience awareness among young writers. The authors recommend taking into consideration individual differences among the students, such as level of self-monitoring and writing profiles (Rijlaarsdam et al., 2009). Interestingly, a second suggestion involves including process measures (in addition to text quality) as dependent variables in intervention studies, a procedure that we adopted in our study (Section Data analysis).

A line of research that is particularly relevant for our study revolves around the development and testing of different types of training interventions (Sato and Matsushima, 2006). For example, López et al. (2017) compared the impact of different strategy-oriented training practices—namely, direct teaching and modeling—on text quality as determined both by readers and by text features. Their modeling training practice involved exposing the students to the think-aloud protocols of an experienced writer while she made a plan for an argumentative text and then wrote down an initial draft. The students were than instructed to emulate the same writing practices. In our study, the same modeling training practice was adopted, but we relied on keystroke logging data rather than on verbalized thoughts (Section Training/intervention). In a follow-up study, López et al. (2021) compared the teaching of explicit revision strategies with a reader-focused condition where students observed a reader interacting with a problematic text and suggesting how the text could be improved. Results showed no difference between the effects of the two training approaches (López et al., 2021). Unlike our cohort of university students, the participants in López et al. (2021) were between 11 and 12 years of age and conducting the revision task in their L1.

Acquiring audience awareness that leads to the production of easy-to-understand texts might be a particularly complex undertaking for L2 writers/revisers, who tend to overly focus on surface-level errors when reading texts to evaluate and to revise them (Traga Philippakos et al., 2018). Low proficiency levels in L2 are particularly detrimental to considerations about the reader and to the development of global revision skills (Barkaoui, 2007). The challenges of revision in L2 have led researchers to investigate students' self-reported abilities to carry out low-level (e.g., grammatical or lexical issues) and high-level text revisions (e.g., text organization) (Chen and Zhang, 2019), along with the cognitive processes that underlie text revision in L2 (Révész et al., 2019). Attention has also been devoted to training that could help L2 writers to independently use and self-regulate writing strategies such as planning or revising content (Chen et al., 2022), as well as to training focusing on texts as global entities that are part of a broader sociocultural context including the reader (Li, 2012; Graham, 2018). Overall, these studies have shed light on the positive effects of revision instruction among L2 writers (Kuteeva, 2011).

### Research gaps and research aims

As this review of prior work has shown, the ability to revise texts in L2 so as to make them comprehensible and usable for the target reader is dependent upon years of writing expertise and language proficiency. In particularly, expert and proficient writers differ from novices by virtue of their non-linear, recursive revision processes and the quality/readability of their texts. Training has been found to help less skilled writers with text simplification tasks. However, several gaps in the research remain to be addressed. First of all, most works so far have focused on writers revising/simplifying their own texts in their L1. Secondly, to the best of our knowledge, research on how text simplification training influences writing phases is lacking. Finally, potential relationships between, on the hand, writing phases during text simplification and, on the other hand, text quality (defined as readability) remain to be investigated.

Therefore, this study addressed the following novel research questions:

- How does text simplification training influence text readability and the dynamics of writing phases in a second language?
- What is the relationship between the pausing dynamics of writing phases and text readability?

Concretely, we first examined the impact of our (online and multimodal) text simplification training (Rossetti and Van Waes, 2022) on product quality (text readability), and combined this with a process-oriented approach to L2 writing by taking into account (potential) differences between writing phases (specifically, first draft and second draft). Secondly, we investigated the relationship between text readability and the students' pausing behavior during different writing phases, when simplifying already existing texts. To the best of our knowledge, no other studies have addressed the same questions.

This focus on considering writing phases—rather than the text simplification process as a whole—is motivated by previous research showing the specificity of each sub-component of the writing process [Sections The (non-) linearity of the writing process and The temporal organization and dynamics of the writing process], as well as by our preliminary findings that text simplification training had limited impact on the cognitive effort of the entire writing process, as indicated by pausing behavior [Rossetti and Van Waes under review (b)].

## Materials and methods

### Participants

The study is based on an experiment involving 47 Master students (96% native speakers of Dutch) from the Faculty of Business and Economics at the University of Antwerp. The students were on average 23 years old, and most of them (80%) indicated *female* as their gender. They had on average between 8 and 9 years of English study, which means that their level of English proficiency was quite high. The majority of them (around 70%) had never taken part in training on text simplification, but 17 participants reported being already familiar with some of the principles of text simplification. With regard to the topic of sustainability, the students assigned a quite low average score of 4 (on a 10-point scale) to their prior knowledge about this topic. We collected data in a laboratory on campus between October and November 2021, after receiving approval from the Ethics Committee for the Social Sciences and Humanities at the University of Antwerp (reference SHW_20_87)^1^.

#### Design and procedure

We adopted a pre-test post-test design, and we randomly divided the students into an experimental and a control group. During the pre-test session, all participants received an extract of a real corporate report (in English) which belonged to a tobacco company fictitiously renamed *SmokIT* (see Section Experimental materials (texts) for details on the texts). The participants were asked to read and revise this extract in order to make it easier to read and engaging for customers, so that it could be published on the company's website. After this pre-test revision task, the students accessed the online modules assigned to them. The experimental group received a link to a module on the simplification of sustainability content, while the module assigned to the control group focused exclusively on the topic of sustainability and contained no instruction on plain language writing or revision (see Section Training/intervention for details on the modules). Students were instructed to spend at least 45 minutes interacting with the theoretical and practical components of their respective modules. The last step of the pre-test session involved completing a short questionnaire that revolved around demographic and background data, namely age, gender, native language(s), years of English study, university programme, prior training and knowledge of plain language principles, prior knowledge about the topic of sustainability, and self-reporting of new knowledge acquired thanks to the modules. The questions were the same for both experimental and control group, with the exception of the questions about plain language, which were only relevant for the experimental group by virtue of the training that they received.

We carried out the post-test session about 2–3 days after the pre-test session. In the post-test session, the students were assigned another extract of a corporate report from the same tobacco company. The task was the same as in the pre-test, namely revising the extract with the aim of rendering it easier to read and more engaging for lay customers searching the website of the company. However, differently from the pre-test, in this session the students were encouraged to use the knowledge gained from their respective modules (Section Training/intervention), and to consult the modules as needed during the revision task. The last step of the post-test session involved the completion of a fidelity questionnaire containing multiple-choice questions about the contents of the modules. The goal of the fidelity questionnaire was to check how much the students had learnt from and engaged with the training materials. Since the modules were different, the experimental and the control group received two different fidelity questionnaires, each tailored to the contents of their respective training. The students were able to answer the majority of the questions correctly (average scores were 0.93 out of 1 for the experimental group and 0.87 out of 1 for the control group), thus showing that they had familiarized themselves with the contents of the modules.

The revision tasks in the pre-test and in the post-test took place in Microsoft Word, while the keystroke logging tool Inputlog was working unobtrusively in the background by recording keyboard, mouse, and Internet search activity (Leijten and Van Waes, 2013). Even though both the pre-test and post-test tasks took place in a laboratory, we tried to create a working environment as relaxed and as ecologically valid as possible. Specifically, the students could carry out online searches, be creative with their revisions, and take as much time as they needed in order to revise the texts.

#### Experimental materials (texts)

The extracts assigned to the students for the revision tasks in the pre-test and in the post-test dealt with different aspects of sustainability so as to avoid a learning effect. The length and the readability level of the original extracts was controlled since these aspects could have acted as confounding variables. Specifically, the two texts were very similar in length (274 vs. 278 words). An analysis with the Coh-Metrix Common Core Text Ease and Readability Assessor (T.E.R.A.) also showed that both texts had a low level of narrativity (which means that they contained complex noun phrases while lacking common words and verbs), a low level of syntactic simplicity, and an average level of referential cohesion, namely an average amount of repetition of words, phrases, and concepts across sentences (Jackson et al., 2016). It should be remembered here that Coh-Metrix provided the data for our readability analysis of the texts revised by the participants (Section Readability data). We also manipulated the original extracts so that they contained the readability issues mentioned in our experimental module, namely issues with vocabulary, syntax, cohesion, relevance, and visual aspects. As mentioned in the Section Design and procedure, students revised these extracts of corporate reports with the goal of making them easier to understand and appropriate for a corporate website. This revision task was informed by previous empirical evidence highlighting the technical and difficult language of sustainability content in corporate reports (Smeuninx et al., 2020).

#### Training/intervention

The text simplification training represents the intervention used in our study. The experimental group received training on the simplification of sustainability content. Specifically, their training material focused on three main sub-topics, namely: principles of accessible communication; sustainability; and text revision. The principles of accessible communication that we introduced dealt with different textual elements, from vocabulary and sentence structure to cohesion, content relevance, and visual aspects. For instance, regarding cohesion, we defined this concept, we provided an example of a cohesive text, and we introduced the revision strategies that a writer can adopt in order to increase the cohesion of texts. The section on sustainability introduced this concept and them zoomed in on the communication of sustainability through reports and websites, in line with the goal of the revision task (section Design and procedure). Finally, the theoretical part of the experimental module ended with a video showing how different levels of expertise are reflected in the processes of text simplification. To this end, we used the Inputlog process graphs obtained from an expert and a less expert writer so that the students could compare the two processes and try to model their revisions to those of the expert (López et al., 2017). Our aim with these graphs was therefore to foster observational learning, which allows students to learn by observing experts at work (Rijlaarsdam et al., 2008). It is important to mention that the Inputlog process graphs represent an important component of our analysis of writing phases and are described in detail in section Criteria for identifying writing phases. A more detailed description of the experimental module is available in Rossetti and Van Waes (2022). The training that the control group received did not contain any reference to text simplification, nor to the revision process. Instead, the content was divided into three main sub-topics related exclusively to sustainability. Specifically, the module explained what sustainability is, how it has evolved over the years, and how it is communicated.

Both types of training were hosted on Calliope, an online writing center (Van Waes et al., 2014) ^2^. In line with the typical structure of the Calliope modules, our training material was also divided into introduction, theory, exercises, and case study. The students could switch freely between theoretical and practical elements, as well as choose to which component they should devote more time, depending on their preferences and learning styles (Van Waes et al., 2014). Additionally, the modules were multimodal as they contained theoretical content in textual and audiovisual formats. A preliminary usability evaluation had shown that multimodality was appreciated by the students (Rossetti and Van Waes, 2022).

#### Data analysis

For this study, we collected data on the text simplification *process* (its phases and related pauses/revisions) using the keystroke logging tool Inputlog 8 (Leijten and Van Waes, 2013). To collect data on the quality of the revision *product* (i.e. text readability), we used the computational tool Coh-Metrix (Section Readability and text quality). In this section, we start by briefly describing how the collected data were prepared prior to analysis. In the case of keystroke logging data, we also outline the criteria that we adopted in order to identify writing phases during text simplification. Due to the richness of data that both Inputlog and Coh-Metrix provide, we selected specific variables to be included in our analysis. Therefore, we outline the selection process of the variables below. Finally, we briefly introduce the types of statistical analyses that we carried out.

##### Data cleaning and filtering

###### Inputlog

For each participant and each session, Inputlog produced a log (IDFX) file, an XML-file containing a linear storage of the keystroke logging data that constitute the basis of the analyses (also carried out by Inputlog). We first produced a general analysis of all IDFX files. In the general analysis, every row corresponds to a different log event (e.g., a key being pressed) for which pause length, pause location, cursor position, environment (e.g., Word document or external source) and evolving length of the document are reported (Leijten and Van Waes, 2013). Thanks to the general analysis, we were able to identify the technical “noise” that had to be removed from the data and to time-filter the IDFX files starting from the moment in which the students opened the Microsoft Word document before making their first revision (Leijten and Van Waes, 2020).

We excluded four participants from the pre-test session and two participants from the post-test session because their IDFX files were irreparably damaged.

###### Coh-metrix

We obtained the readability scores automatically using the desktop version of Coh-Metrix, which allowed us to upload and to simultaneously process more than one text (McNamara et al., 2014). The majority of participants −90% in the pre-test session and 73% in the post-test session—rewrote the assigned text from scratch; the others revised the text assigned itself [Rossetti and Van Waes, under review (b)]. Therefore, in some of the final Microsoft Word documents, the original and the rewritten texts were both present. In these cases, prior to using Coh-Metrix, we deleted the original text so as to ensure that only the rewritten—simplified text—remained in the Microsoft Word document and would be analyzed by Coh-Metrix.

##### Keystroke logging data

###### Criteria for identifying writing phases

In this study, our goal was to identify two main writing phases in the process of text simplification, namely a first phase during which a first “stable” draft of a simplified text is produced (either rewritten from scratch or revised from top to bottom), and a second phase during which the first draft is re-read and, if necessary, revised again. As such, this study did not adopt a fine-grained view of non-linearity [as was the case in Baaijen and Galbraith (2018), for instance, who considered all actions preceding the production of new text], but rather a global, macro-level view of non-linearity. Furthermore, rather than using the traditional, time-based distinction between planning, formulation, and revision of content [see e.g., Beauvais et al. (2011)], we relied on keystroke logging data about writing dynamics. Xu and Xia (2021) had already made a step in this direction by using the movement of the cursor position toward the beginning of the text as the start of a revision/reviewing phase. In this study, we expanded on the (visual) criteria and defined a combination of writing dynamics that could be used to distinguish between writing phases during text simplification.

**Figure 1:**
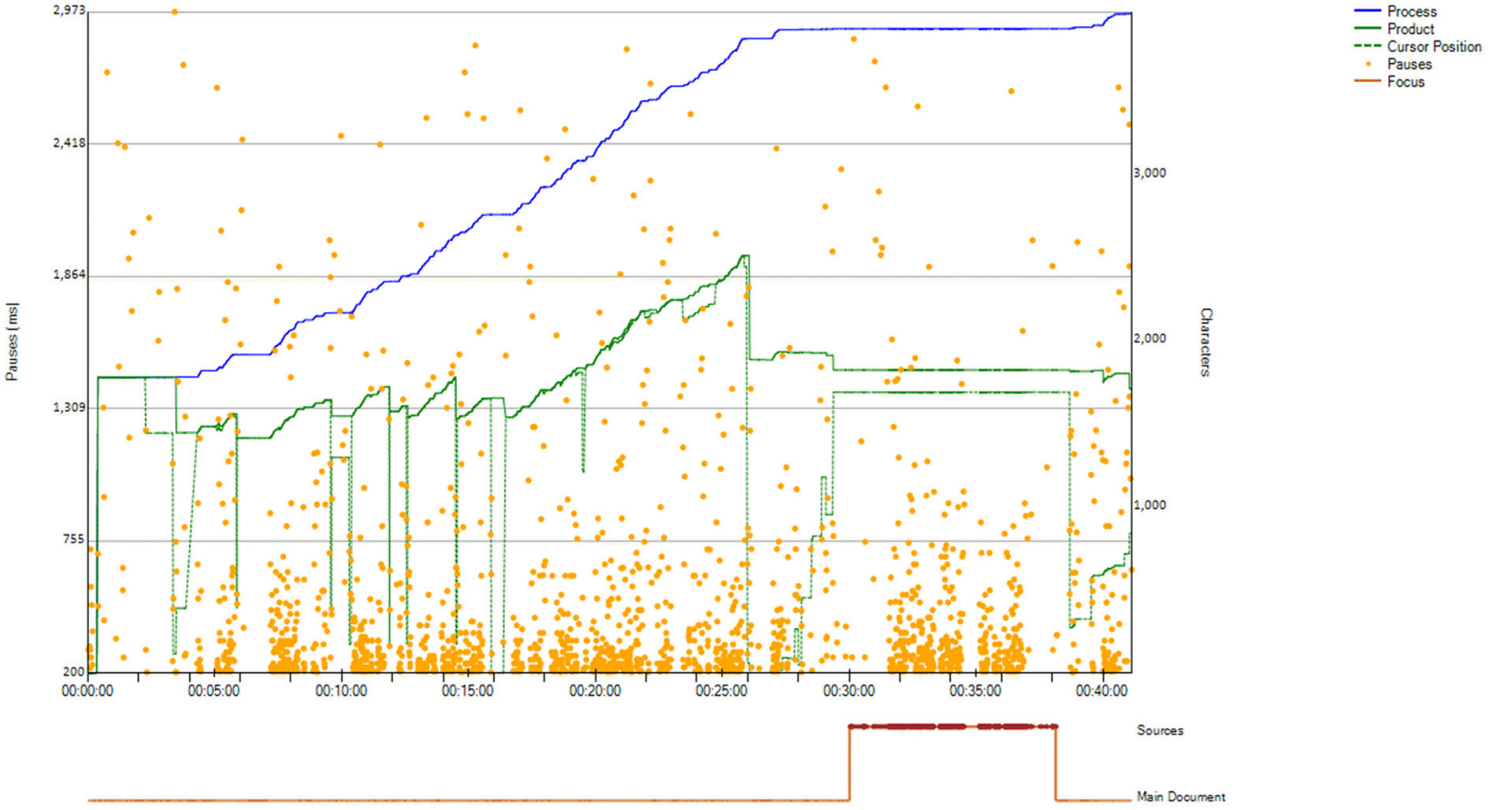
Example of an Inputlog process graph.

Concretely, we first generated process graphs for all the IDFX files. Figure 1 below is an example of an Inputlog process graph. The process graphs are visual representations of the writing process that report data on: length of a text (number of characters) at any given point (green product line); number of characters added/modified/deleted (blue process line); location and duration of pauses (orange dots); position of the cursor (dotted green line); use of sources (orange line below the graph); and overall task duration (x axis) (Vandermeulen et al., 2020).

Following independent examination of a sub-set of process graphs, the two authors discussed the criteria that could be used to identify the pivot point between the first and the second phase, and they agreed to use all of the components of a process graph [differently from Xu and Xia (2021), who relied exclusively on the cursor position]. The development and testing of the criteria used is beyond the scope of this article. However, in the interests of clarity, below we report a summary of the selected criteria:

- The product (green, continuous) line flattens, indicating that production of new text has slowed down/stopped and that re-reading of the existing draft has become the dominating strategy;
- The distance between the product (green, continuous) line and the process (blue) line increases, indicating that the reviser has deleted unnecessary text from the first draft and is ready to start focusing exclusively on a second draft;
- The cursor (green dotted line) moves toward the start of the text, indicating that the reviser is about to check the entirety of their draft from top to bottom as part of a reflective second phase;
- The interaction with sources (line below the graph) is minimized as the reviser's focus switches from consulting external sources for new text production to checking the existing draft itself;
- Longer pauses become more frequent, as the predominant activity in the second phase is reading the text produced up to that moment.

It should be mentioned that not all of the criteria were relevant for all of the process graphs. Furthermore, for six participants, the authors agreed that no second phase was initiated (cf. *first-draft-final* approach).

###### Pausing and revision variables

We used the filtered IDFX files (section Inputlog) to generate the pause analyses and the revision analyses in Inputlog. For the pause analysis, we used a threshold of 200 milliseconds as shorter pauses are mainly linked with transition between keys rather than with cognitively demanding processes (Van Waes et al., 2009). In addition to data on general pausing behavior (e.g., total number of pauses), we focused on pauses at multiple text levels (word, sentence, and paragraph levels) so as to gain a broad understanding of the cognitive effort involved in text simplification. In the case of revision, we calculated the events and the number of characters involved in three types of operations, viz. addition of new text at the end of the text produced so far, insertions, and deletions. After generating the pause analysis and the revision analysis for each participant individually, Inputlog allowed us to merge the output files from multiple analyses and to analyze them in an aggregated way.

#### Readability data

As explained in the Section Readability and text quality, Coh-Metrix is a computational tool that—by applying Natural Language Processing and Latent Semantic analysis—provides multiple indices of text readability mainly related to words, syntax, discourse, and cohesion (Graesser et al., 2014). It also provides results from two traditional readability formulas (the Flesch-Kincaid Grade Level and the Flesch Reading Ease). For the purposes of this study, and in order to gain a broad perspective of the level of readability that the students achieved in their texts, we selected Coh-Metrix indices related to: overall text length; word length and word familiarity; sentence length/structure/similarity; referential cohesion; and deep cohesion. Additionally, we reported results from traditional readability formulas.

The notions of referential cohesion and deep cohesion might need some explanation. Referential cohesion can be defined as the relatedness between ideas in a text, and it can facilitate reading comprehension especially for low-knowledge readers (McNamara et al., 2010). Three indices of referential cohesion that were of special interest for this study were: noun overlap (i.e., the repetition of the same noun, in the same morphological form, between sentences); argument overlap (namely, the repetition of the same pronoun or noun, in a different morphological form, between sentences); and stem overlap, occurring when a word or a pronoun has the same lemma as a word in another sentence (McNamara et al., 2010). Deep cohesion is determined by the amount of connectives (e.g., temporal, causal, logical) that link sentences and ideas together, thus helping the reader build a deep understanding of the text (Dowell et al., 2016).

#### Statistical analyses

We carried out our statistical analyses in SPSS, mainly using variance and correlation analyses. When the data were not normally distributed, we relied on non-parametric tests (Mann-Whitney U test and Wilcoxon Signed-Rank test). Finally, it should be noticed that, when comparing the first and the second phase of revision (section Writing phases during text simplification), and when conducting the correlation analyses (Section Correlations between pausing during text simplification and text readability), we maintained the distinction between pre-test and post-test, but merged the data from the experimental and the control group.

## Results

In this section, we first present the differences between writing phases of text simplification using data from all the participants, so as to provide evidence of the validity of our phase-classification criteria (Section Criteria for identifying writing phases). We then report the results related to the impact of our text simplification training on pausing and revision during writing phases and on text readability, respectively. Finally, we present the findings of the correlation analyses between writing process characteristics (i.e. pausing during both phases) on the one hand, and product quality (i.e. text readability) on the other hand.

### Writing phases during text simplification

**Figure 2:**
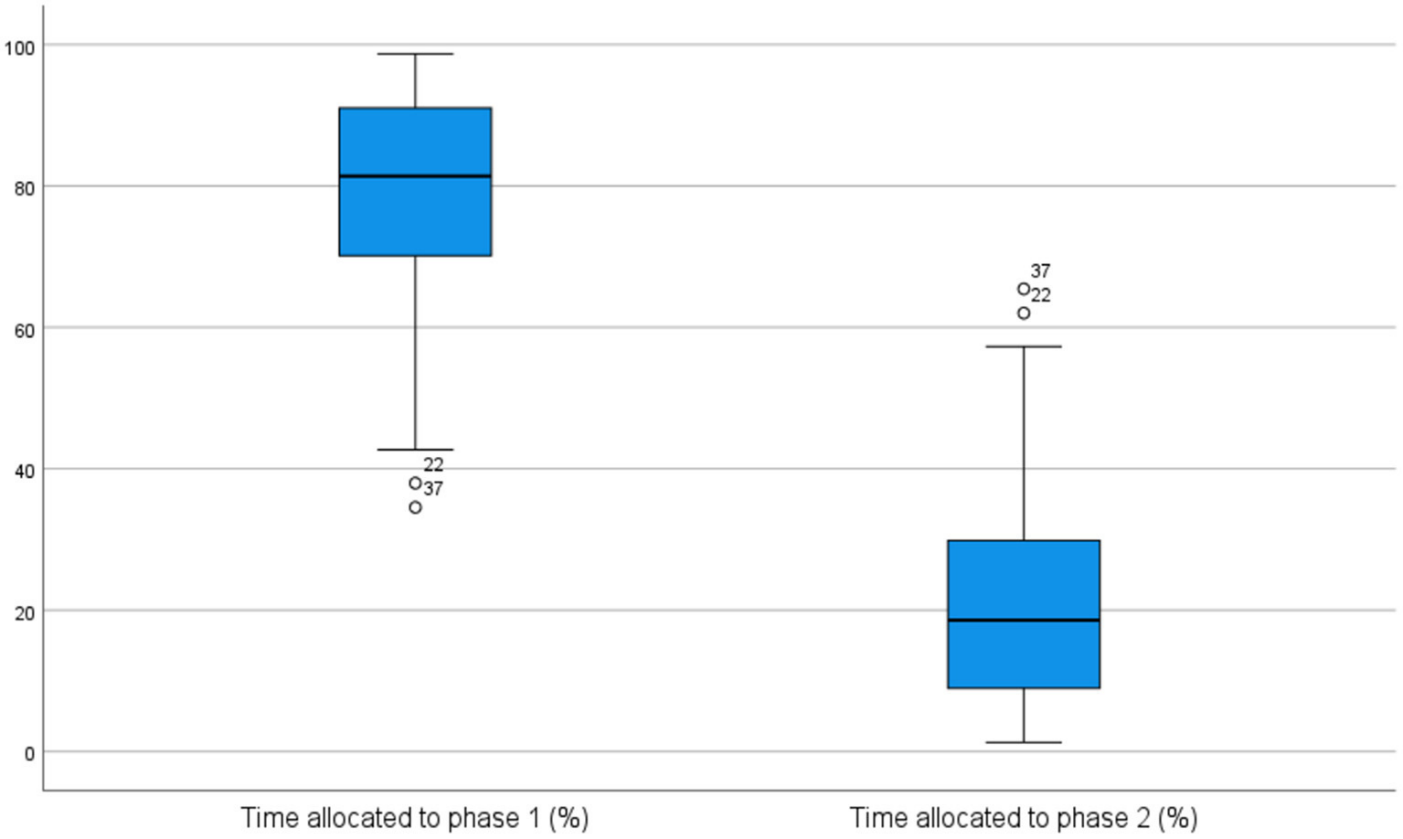
Time distribution across phases in pre-test session.

**Figure 3:**
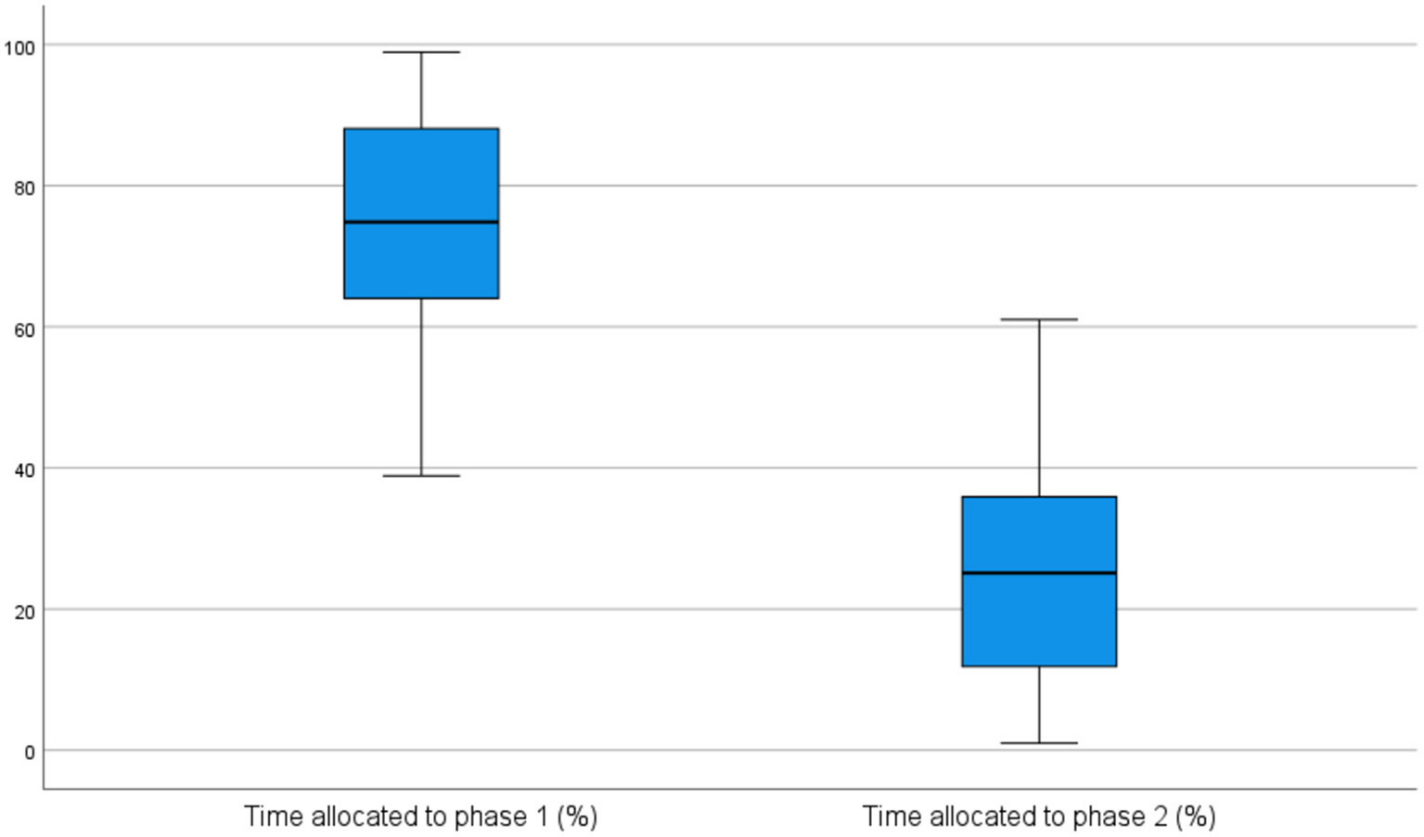
Time distribution across phases in post-test session.

On average, in the pre-test session, the students spent about 79% of the overall process time in the first phase and about 21% of the overall process time in the second phase. The difference in time allocation to each phase was statistically significant, as indicated by the Wilcoxon Signed-Rank test (*z* = −5.275, *p* = <0.001), and was also reflected in the distribution of their pauses. Concretely, 80% of the total number of pauses occurred during the first phase, and 20% in the second phase. In the post-test session, the distribution of time and pauses allocated to each phase was quite similar to the pre-test distribution. Specifically, the first phase occupied about 73% of the overall process time and included about 75% of the total number of pauses, with the differences between phases being again statistically significant (*p* = <0.001). Figures 2, 3 visually report the distribution of time across phases in the pre-test and in the post-test session, respectively.

In order to examine the extent to which the two phases differed with respect to the process characteristics defined, we ran Wilcoxon Signed-Rank tests comparing the first phase and the second phase in both pre-test and post-test session. The results are reported in Table 1, where significance level refers to the comparison between phase 1 and phase 2. Overall, the analysis reveals quite some major differences in cognitive activity between the two phases. Specifically, both in the pre-test and in the post-test, the first phase was significantly longer (including longer pause time and longer active writing time) than the second phase. Unsurprisingly then, the total number of pauses and the number of P-Bursts—namely, bursts of writing interrupted by a pause of at least two seconds (Baaijen et al., 2012)—were also significantly higher in the first phase. These differences were reflected at all pause location levels (within words, before words, before sentences, before paragraphs, before revisions, between words, and between sentences).

**Table 1:** Pausing behavior across writing phases.

**Pause variables**	**Phase**	**Pre-test**			**Post-test**		
		**Mean**	**SD**	***p***	**Mean**	**SD**	***p***
Total process time	*1*	0:33:41	0:14:16	<0.001^*^	0:36:59	0:15:30	<0.001^*^
	*2*	0:09:58	0:08:53		0:12:45	0:08:16	
Total pause time	*1*	0:22:44	0:10:39	<0.001^*^	0:24:42	0:10:34	<0.001^*^
	*2*	0:06:18	0:05:33		0:07:56	0:05:27	
Total active writing time	*1*	0:10:56	0:05:13	<0.001^*^	0:12:17	0:06:10	<0.001^*^
	*2*	0:03:38	0:03:42		0:04:48	0:03:22	
Proportion of pause time	*1*	67%	10%	0.10	67%	7%	<0.05^*^
	*2*	64%	10%		62%	12%	
Total number of pauses	*1*	1,159	518	<0.001^*^	1,311	614	<0.001^*^
	*2*	314	313		413	296	
Mean pause time (secs)	*1*	0.55	0.06	<0.05^*^	0.54	0.07	0.05
	*2*	0.59	0.12		0.58	0.11	
Median pause time (secs)	*1*	0.42	0.06	<0.05^*^	0.42	0.68	<0.05^*^
	*2*	0.48	0.10		0.47	0.09	
Number P-Bursts	*1*	115.33	50.85	<0.001^*^	127.83	54.40	<0.001^*^
	*2*	32.41	27.14		41.08	29.89	
Number within-word pauses	*1*	354.36	203.59	<0.001^*^	401.03	225.01	<0.001^*^
	*2*	76.05	87.51		102.83	92.82	
Mean within-word pauses	*1*	0.32	0.02	1.00	0.32	0.02	0.66
	*2*	0.33	0.04		0.33	0.06	
Number before-word pauses	*1*	168.92	81.51	<0.001^*^	181.93	88.08	<0.001^*^
	*2*	37.92	53.22		44.90	37.59	
Mean before-word pauses	*1*	0.57	0.14	0.10	0.54	0.12	0.30
	*2*	0.53	0.23		0.51	0.17	
Number before-sentence pauses	*1*	9.49	7.53	<0.001^*^	11.23	7.37	<0.001^*^
	*2*	1.82	2.83		3.33	3.36	
Mean before-sentence pauses	*1*	0.61	0.39	1.00	0.69	0.87	0.001^*^
	*2*	0.46	0.22		0.34	0.34	
Number before-paragraph pauses	*1*	12.46	8.23	<0.001^*^	14.38	8.36	<0.001^*^
	*2*	3.54	5.01		4.33	4.63	
Mean before-paragraph pauses	*1*	0.61	0.20	<0.01^*^	0.65	0.34	<0.05^*^
	*2*	0.36	0.32		0.47	0.30	
Number revision pauses	*1*	131.82	82.67	<0.001^*^	151.57	95.44	<0.001^*^
	*2*	38.97	48.76		45.25	42.67	
Mean revision pauses	*1*	0.57	0.09	0.51	0.55	0.79	0.10
	*2*	0.53	0.17		0.53	0.15	
Number between-word pauses	*1*	220.23	112.83	<0.001^*^	255.50	122.08	<0.001^*^
	*2*	48.59	82.85		65.40	63.98	
Mean between-word pauses	*1*	0.57	0.13	0.87	0.55	0.13	<0.05^*^
	*2*	0.57	0.20		0.49	0.17	
Number between-sentence pauses	*1*	7.46	4.63	<0.001^*^	8.82	5.77	<0.001^*^
	*2*	1.44	4.10		2.45	2.98	
Mean between-sentence pauses	*1*	0.83	0.55	0.72	1.07	0.96	<0.05^*^
	*2*	0.65	0.64		0.58	0.53	

The * symbol indicates statistical significance at the *p* value <0.05.

The second phase, in contrast, was characterized by less frequent pauses but with an overall longer average pause time (as indicated by the longer mean and median pause times). This result is consistent with the reading behavior that is typical of a second/reviewing phase, during which writers re-read and re-check their semi-final drafts from multiple perspectives, while producing less ‘new' text (Perrin and Wildi, 2008; Xu and Xia, 2021). Taken together, these results indicate that the first phase of the text simplification process was more elaborated that the second phase, and that the phases were characterized by diverging cognitive processes. These differences that we observed in terms of pausing behavior between the first and the second phase of text simplification are also methodologically interesting, as they provide some empirical validation of the criteria that we used in order to identify (re)writing phases (Section Criteria for identifying writing phases).

When focusing specifically on the differences between pre- and post-test, it can be observed that, in the post-test exclusively, the average duration of the before-sentence pauses, the average duration of the between-sentence pauses, and the average duration of the between-word pauses appeared to be significantly higher in the first phase than in the second phase. These results are interesting when considering that the experimental group produced syntactically simpler texts, with shorter words, in the post-test session (Section Impact of text simplification training on text readability). In the pre-test session, the average duration of the pauses was not significantly different, with the exception of before-paragraph pauses, whose average duration was significantly higher (almost double) in the first phase.

With regard to revision behavior, Table 2 reports the results of the Wilcoxon Signed-Rank tests comparing the first phase and the second phase in both pre-test and post-test session (therefore, significance level refers here again to the direct comparison between the two phases). It can be observed that the two phases show again a number of significant differences, and an interesting pattern emerges when comparing pre-test with post-test. Concretely, in the pre-test session, the second phase was characterized by a significantly higher number of events (occurrences) of revisions, including normal production (i.e. new text being added at the end of the existing draft), insertions, and deletions events. On the other hand, the first phase was characterized by a significantly higher number of characters being revised (inserted and deleted). These differences seem to indicate that, in the first phase of the pre-test, the students' revision behavior involved more substantial revision sessions, while in the second phase, the students' changes were more frequent but less substantial.

**Table 2:** Revision behavior across writing phases.

**Revision variables**	**Phase**	**Pre-test**			**Post-test**		
		**Mean**	**SD**	***p***	**Mean**	**SD**	***p***
Count of all revision events	*1*	250.49	139.66	<0.001^*^	254.33	129.69	<0.001^*^
	*2*	996.72	1,140.45		85.47	85.19	
Sum of characters in all revision events	*1*	7,858.49	7,509.93	<0.001^*^	6,683.35	5,136.26	0.001^*^
	*2*	4,064.23	8,223.70		3,869.72	5,208.27	
Normal production (events)	*1*	41.86	49.56	<0.001^*^	51.14	49.97	<0.05^*^
	*2*	304.54	331.33		17.94	20.05	
Normal production (characters)	*1*	2,106.70	1,723.78	0.057	1,817.69	1,517.51	0.549
	*2*	1,256.60	1,546.09		1,497.94	1,912.41	
Insertion (events)	*1*	95.87	80.95	<0.05^*^	86.60	71.37	<0.05^*^
	*2*	390.84	686.37		38.33	47.99	
Insertion (characters)	*1*	2,460.26	2,488.15	<0.05^*^	2,504.55	2,218.24	<0.05^*^
	*2*	1,846.81	5,436.73		1,227.76	1,790.80	
Deletion (events)	*1*	117.36	66.12	<0.001^*^	119.79	62.92	<0.001^*^
	*2*	402.72	431.97		41.02	40.17	
Deletion (characters)	*1*	3,462.64	6,191.19	<0.001^*^	2,461.63	2,708.57	<0.05^*^
	*2*	1,421.18	3,220.55		1,673.40	2,899.75	

The * symbol indicates statistical significance at the *p* value <0.05.

The post-test session shows a different pattern. Concretely, the number of revision events and the number of characters being revised were both significantly and consistently higher in the first phase than in the second phase.

### Impact of text simplification training on writing phases

Following evidence that the first phase and the second phase of text revision required different levels of cognitive effort and involved a different revision behavior, we turned our attention to how these phases differed as a result of our text simplification training. We focused in particular on differences in pausing behavior and revision behavior. It should be remembered that a preliminary analysis of the overall text simplification process had shown no significant results between experimental and control group in terms of pausing behavior [Rossetti and Van Waes, under review (b)]. In this article, rather than considering the text simplification process as a whole, we divide it into phases.

With regard to pausing behavior, we used a broad range of pause-related variables, including those reported in Table 1, as dependent variables. We carried out a two-way MANOVA in order to examine whether the differences in pauses that we observed between phases were dependent on the text simplification training that the participants received. We found no statistically significant interaction effect between writing phases and text simplification training (i.e. the intervention) on the combined pause-related dependent variables, [*F*_(32, 17)_] = 0.804, *p* = 0.712; Wilks' Λ = 0.398.

As far as revision behavior is concerned, we used the same revision-related variables reported in Table 2. We again carried out a two-way MANOVA in order to examine whether the differences in revisions that we observed between phases were dependent on the text simplification training. Similar to the pausing behavior, we found no statistically significant interaction effect between writing phases and text simplification training on the combined revision-related dependent variables, [*F*_(6, 58)_] = 0.216, *p* = 0.970; Wilks' Λ = 0.978.

### Impact of text simplification training on text readability

Overall, following text simplification, the average text length was 288 words during the pre-test session and 293 words during the post-test session. When examining differences in the readability level achieved by the experimental (*N* = 23) and control group (*N* = 19) during the pre-test session (i.e. prior to training) using the Mann-Whitney U test, we found no significant differences (*p* > 0.05). However, after taking part in our training, the experimental group produced texts that had fewer words (average length was 270 words compared with an average length of 320 words from the control group, *z* = −2.161, *p* = 0.03), contained shorter sentences (average sentence length was 14.93 words compared with 17.28 words in the control group, *z* = −2.767, *p* = 0.006) that were also syntactically simpler (average syntactic simplicity score was 50.71, compared with an average score of 39.49 for the control group, *z* = −2.439, *p* = 0.015), and could be read by people with fewer years of education, as indicated by the Flesch-Kincaid Grade level measuring word length and sentence length (the average Flesch-Kincaid Grade level was 9.85 for the experimental group and 10.86 for the control group, *z* = −2.515, *p* = 0.012). Interestingly, in the post-test session, the control group produced texts with higher argument overlap between adjacent sentences (average score for the control group was 0.58 vs. 0.47 for the experimental group, *z* = −1.973, *p* = 0.049), which is one of the indices of referential cohesion. This result might be due to the topic-centered training that the control students received (Section Training/intervention), which might have led them to consistently repeat the same nouns linked with sustainability across sentences.

### Correlations between pausing during text simplification and text readability

We used Pearson correlations to investigate potential relationships between, on the one hand, pausing behavior across phases during text simplification and, on the other hand, text readability as assessed through Coh-Metrix. In the interests of clarity, and due to the high number of readability and pause-related variables, here we will not discuss weak or negligible relationships. With regard to pausing behavior, we delve in particular into the pauses at different text levels, viz. the word-, sentence-, and paragraph level, as well as pauses preceding revisions. Pauses located *before* and *between* words, as well as pauses located *before* and *between* sentences, showed strong to moderate correlations (6 < *r* <1). Therefore, when the correlation results coincided, we chose to report results related exclusively to between-level pauses so as to avoid repetitions, and guided by the notion that transition times between text levels are more representative of underlying cognitive processes (than before- or after- times) (Baaijen et al., 2012). However, when moderate or strong correlations were observed exclusively for the *before* pauses, we reported them as well.

#### Pre-test session

##### Phase 1

Overall, the correlation analysis showed that different aspects of readability each correlated with different levels of pause location. We started by examining *within-word pauses*, which were likely caused by the cognitive effort of using spelling and orthography in L2 (Rønneberg et al., 2022). In the first phase of the pre-test, there was a moderate, negative, and significant relationship between the median time of within-word pauses and referential cohesion (*r* = −0.396), including its indices of noun overlap (*r* = −0.350), stem overlap (*r* = −0.319), argument overlap (*r* = −0.422), and content word overlap (*r* = −0.343) across all sentences. In contrast, referential cohesion (*r* = 0.325) and deep cohesion (*r* = 0.336) appeared to be moderately, positively, and significantly correlated with the number of *between-word pauses*.

At the sentence level, the proportion of *between-sentence pauses* (*r* = 0.340) were moderately, positively, and significantly correlated with syntactic simplicity.

At the paragraph level, the average duration of *before-paragraph pauses* was moderately, positively, and significantly correlated with syntactic simplicity (*r* = 0.330), but negatively correlated with argument overlap across all sentences (*r* = −0.315), one of the indices of referential cohesion.

Finally, we found a moderate, positive, and significant correlation between proportion of *revision pauses* and level of referential cohesion (*r* = 0.309), which might indicate that, the more the students revised (deleted/inserted content), the more they increased the amount of idea repetitions within the text.

##### Phase 2

The correlations between readability indices and pausing behavior shifted to some extent in the second phase. For instance, the number of *within-word pauses* (*r* = −0.356), the number of *before-word pauses* (*r* = −0.348), and the number of *before-sentence pauses* (*r* = −0.356) were all moderately, significantly, but negatively correlated with the Flesch-Kincaid Grade level, which assigns a grade level matching the difficulty of a text (the lower the score, the easier the text), mainly based on word length and sentence length (Crossley et al., 2017). Furthermore, the mean duration of *before-word pauses* was moderately, significantly, and positively correlated with narrativity, which measures word familiarity and everyday vocabulary (*r* = 0.384).

With regard to the sentence level, we found the most outspoken, positive, and significant correlations between the mean duration of *before-sentence pauses* and sentence syntax similarity between adjacent sentences (*r* = 0.637) and across paragraphs (*r* = 0.641).

Considerations at the paragraph level also seemed to play a more important role in the second phase compared with the first phase, possibly because—having produced a fully formed draft—the students were able to look at their texts as whole entities. Specifically, the median duration of *before-paragraph pauses* was strongly, positively, and significantly correlated with argument overlap between adjacent sentences (*r* = 0.580) (while, in the first phase, the duration of these pauses and argument overlap were negatively correlated). However, the median duration of before-paragraph pauses continued to be negatively correlated with two indices of referential cohesion, namely noun overlap across all sentences (*r* = −0.444) and stem overlap across all sentences (*r* = −0.590).

The results on before-paragraph pauses are confirmed by the results on *revision pauses* as their average duration was also moderately, negatively, and significantly correlated with noun overlap across all sentences (*r* = −0.431) and with stem overlap across all sentences (*r* = −0.497). The students did, however, gave prominence to syntactic simplicity during their revisions as this readability measure correlated moderately and significantly with the number of revision pauses (*r* = 0.381).

#### Post-test session

##### Phase 1

The correlations between readability indices and pausing behavior shed light on interesting patterns also when examining the post-test session. In the first phase of the post-test, the geometric mean of *within-word pauses* was negatively correlated with one of the indices of referential cohesion (i.e. argument overlap between adjacent sentences) (*r* = −0.320). With regard to *between-word pauses*, their number was positively, moderately, and significantly correlated with referential cohesion (*r* = 0.394) and with one of its indices, i.e. content word overlap across all sentences (*r* = 0.318). Interestingly, the median time of between-word pauses was negatively correlated (*r* = −0.327) with the Flesch Reading Ease, the traditional readability formula that measures word length and sentence length.

At the sentence level, we found the number of *between-sentence pauses* to be moderately, positively, and significantly correlated with deep cohesion (*r* = 0.397), but not correlated with syntactic simplicity.

At the paragraph level, the median duration of *before-paragraph* pauses was moderately, significantly, and negatively correlated with the level of deep cohesion (*r* = −0.344), while the geometric mean of the before-paragraph pauses was positively correlated with the level of narrativity (*r* = 0.338).

Finally, regarding *revision pauses*, we did not find any (moderate nor strong) correlation with readability measures, differently from what we observed during the first phase of the pre-test.

##### Phase 2

The median time of *between-word pauses* was negatively correlated with both syntactic simplicity (*r* = −0.363) and with deep cohesion (*r* = −0.353). This result suggests that difficult lexical choices requiring more cognitive effort prevented the students from evaluating and revising sentences and their connections.

At the sentence level, the number of *between-sentence pauses* was positively, significantly, and moderately correlated with syntactic simplicity and with sentence syntax similarity across adjacent sentences.

Syntactical considerations seem to have been prominent even when the students paused at the paragraph level. Specifically, the number (*r* = 0.390) and the median duration of *before-paragraph pauses* (*r* = 0.492) were positively, moderately, and significantly related with syntactic simplicity. On the other hand, the number and the median duration of before-paragraph pauses were moderately and negatively correlated with multiple indices of referential cohesion, viz. noun overlap across all sentences (*r* = −0.357), stem overlap across all sentences (*r* = −0.369), and argument overlap between adjacent sentences (*r* = −0.390).

Finally, while in the first phase there were no moderate nor strong correlations with the *revision pauses*, in this second phase the number of revision pauses appeared to be moderately, positively, and significantly correlated with syntactic simplicity (*r* =0.367) and with sentence syntax similarity between adjacent sentences (*r* = 0.355).

## Discussion

In this study, we examined the impact that online and multimodal training on text simplification has on the readability of texts and on the dynamics of the writing (text simplification) process. Furthermore, we investigated the relationship between text readability (product) and pausing dynamics during phases of text simplification (process). In addition to this evaluative perspective, we used the keystroke logging data to describe the participants' writing dynamics as such (e.g., related to the distribution of pauses and revisions across simplification phases). Our focus was on L2 writing as the university students who participated in this study were asked to simplify texts in their L2 (English). The texts reported on a company's sustainability efforts. The students had quite a high level of English proficiency and—even though they were not professional writers—they could be considered as semi-experts since they were enrolled in a Master program in the domain of business communication. This study sought to address different research gaps in terms of writing activity examined (i.e. text simplification of already existing texts in L2), genre of the texts (namely, sustainability reports), and methodological approach (i.e. use of keystroke logging data descriptively, to identify phases of writing, and more evaluatively, in correlation analyses between process- and product-oriented data). In this section, we discuss the findings and the theoretical, methodological, and pedagogical implications of our study. We then conclude by discussing limitations and avenues for future research.

### Discussion of the results

We found that, by taking into consideration multiple writing dynamics (namely, cursor movements, intensity of new text production, decisions about deleting/inserting, duration of pauses, and frequency of interaction with external sources), it is possible to identify two main, high-level writing phases in the text simplification process. These two phases showed differences in terms of the (cognitive) activities involved, with the first phase being longer and predominantly characterized by more frequent pauses at multiple text levels (from words, to sentences, to paragraphs). The average duration of pauses at multiple text levels was also usually higher in the first phase, especially during the post-test session. Furthermore, during the first phase, the students made more substantial revisions (specifically, insertions and deletions) with the aim of simplifying the assigned text. These findings should be interpreted by keeping in mind that the majority of students opted for rewriting a new text from scratch rather than revising the assigned text [Rossetti and Van Waes, under review (b)]. In other words, writing a new, simplified text (in L2) required more effort on the part of students since they had to plan and select vocabulary, sentence connections, paragraph structure, and so on, thus pausing more frequently and, often, for a longer time. As argued in Xu and Qi (2017), pausing is a strategy that writers adopt—consciously or unconsciously—to free up attentional resources that can then be used to solve specific problems. In our study, for instance, longer between-word and between-sentence pauses might indicate that the students gave more careful consideration to issues of sentence structure and vocabulary in the first phase of text simplification (Leijten et al., 2019a). In contrast, the second phase was shorter and characterized by less frequent pauses, but with an overall longer average pause time, which seems to indicate that the students were mainly making limited revisions based on longer episodes of re-reading and re-checking their drafts, as was also observed in other studies (Perrin and Wildi, 2008; Xu and Xia, 2021; Valenzuela and Castillo, 2022).

Some interesting patterns also emerged when comparing the pre-test and the post-test session. In the pre-test session (i.e. before receiving the text simplification training), the students revised the assigned text more frequently (though less substantially) during the second/reading phase. In the post-test session, both the frequency of revisions and the number of characters revised was higher in the first phase. In other words, during the post-test session, the students carried out most—and the most substantial—revisions before finishing a first draft. Even though the activity of revision is the same, the phase during which it occurs might determine its purpose (Van den Bergh et al., 2016). If we refer back to the literature on writing profiles [Section The (non-) linearity of the writing process], this revision behavior seems to be partly similar to that of *fragmentary stage I writers*, who make most revisions while producing a first draft, thus having a fragmented, less linear, and recursive writing process (Van Waes and Schellens, 2003). It is possible that participating in the training (and/or conducting a text simplification task for the second time within a few days) influenced the way in which the students managed their revisions as they had acquired more diverse perspectives on text simplification. However, future research is needed to confirm that hypothesis (Section Limitations and future research), especially considering that we found no statistically significant interaction effect between writing phases and text simplification training (i.e. the intervention) on revision and pausing dynamics.

This lack of observable impact of our text simplification training on pausing and revision dynamics across writing phases might be due to the fact that the students' exposure to the training was fairly limited (i.e. about 45 minutes), or to the fact that a few students (though a minority) were already familiar with some of the principles of text simplification (pre-knowledge). In general, however, modifying the way in which writers organize/manage their processes is a complex and not straightforward undertaking that might require several years and that goes hand in hand with the development of writing expertise in general. For instance, research from Beauvais et al. (2014) showed that, contrary to expectations, intermediate (grade 5) and junior high (grade 7) students did not change the amount of time spent pre-planning content as a result of task complexity. In contrast, grade 9 students spent more time pre-planning when the task was more cognitively demanding. The fact that the students were simplifying a text in their L2 might also explain why the differences between phases in terms of pausing and revision dynamics were fairly consistent before and after the text simplification training. In relation to this point, Van Weijen (2009) found the organization of the sub-processes of writing to be more consistent in L2 (than in L1) across tasks.

Our text simplification training had an impact—though limited—on the readability of the simplified texts. Specifically, after taking part in the training, the experimental group produced texts that contained fewer and shorter words, and shorter and simpler sentences. These results suggest that the students in the experimental group mainly focused on the sections in our training dealing with vocabulary and sentence structure (Section Data analysis) and, in turn, mainly implemented revisions at the word- and at the sentence-level. These types of localized revisions are less cognitively demanding than whole-text revisions addressing cohesion or structure (Piolat et al., 2004). These findings are therefore in line with what had been observed in previous research on the cognitively demanding L2 writing and suggest that, in order to address macro-level issues, even high-language proficiency students might need specific feedback on their writing (Tuzi, 2004).

Our fine-grained correlation analysis also shed light on specific patterns. Concretely, we observed that the number and duration of pauses occurring at specific text locations tended to be linked with increased readability in the same or adjacent text locations, and with decreased readability in other text locations. In particular, longer pausing times caused by low-level issues of spelling and orthography in L2 were related with an inability to move beyond the individual word and to increase cohesion at the global text level (Rønneberg et al., 2022). On the other hand, a higher number of considerations about lexical choices was linked with lower word length and lower sentence length [in line with traditional views of readability (Section Readability and text quality)], higher sentence connectivity, and an increase in the overlap of ideas across sentences. This indicates that, as students were planning which lexical choices to adopt, they tried to simplify vocabulary and sentence structure, they used vocabulary from nearby sentences, and they considered the links between the sentences, thus moving partly beyond the individual sentence level (Graesser et al., 2014). Furthermore, the duration of pauses linked with lexical choices went hand in hand with higher word familiarity. However, lexical choices requiring more cognitive effort (i.e. longer pausing times) seemed to prevent the students from evaluating and revising sentences and their connections. A higher incidence of pauses between sentences was related with syntactic simplicity, which means that, the more our participants considered the formulation of sentences, the more they managed to make them shorter and less convoluted. The number and the average duration of pauses before paragraphs was also linked with higher syntactic simplicity, but with lower aspects of cohesion. In other words, it seems that, as students planned and checked the paragraphs of their texts, they focused mainly on sentence structure, rather than on the overlap of ideas across sentences (especially sentences that were not adjacent).

The correlation analysis also shed light on some differences between phases. For instance, when pausing between sentences in the first phase of the pre-test session, the students mainly focused on making syntactic structures simpler, while in the second phase they focused on making the style and form of texts consistent (McCarthy et al., 2007), thus showing somehow a strategic management of the writing process. Another difference emerged when we observed the pauses preceding revisions. During the first phase of the pre-test session, a higher incidence of these pauses was linked with higher cohesion, which might indicate that, the more the students revised (deleted/inserted content), the more they increased the amount of idea repetitions within the text. In contrast, during the second phase of the pre-test session, the more the participants paused before implementing deletions, additions, and insertions of content, the more they seemed to decrease referential cohesion (namely, the repetitions of ideas). This finding might indicate that, after producing a “stable” first draft, the students tried to make their texts more lexically sophisticated—possibly also by relying on external sources—but additional research is needed to confirm this hypothesis (Section Limitations and future research).

Finally, the correlation analysis also shed light on some differences between pre-test and post-test session. In the post-test session, longer considerations about lexical choices did not seem to consistently revolve around word length. In relation to this point, it should be mentioned that, in the vocabulary section of our training (Section Training/intervention), we explained that word length cannot always be used as a proxy for word difficulty. Furthermore, after taking part in our training, the longer the students examined their sentences, the more they added connectives between them, thus increasing cohesion. Additionally, during the second phase of the post-test session, the number of pauses preceding sentences and the number of pauses preceding revisions were linked with changes that made the sentences both simpler and more similar (while simplicity and similarity were addressed in two separate phases during the pre-test session). We can tentatively assume that our training partly influenced the students' management of revisions during text simplification, and the way in which they approached readability at the word- and at the sentence-level differently, but again additional research is needed (Section Limitations and future research).

### Theoretical, methodological, and pedagogical implications

This study has theoretical, methodological, and pedagogical implications. From a theoretical point of view, one of the most up-to-date cognitive models of writing identifies three main levels influencing the writing process, namely a control level, a process level, and a resource level (Hayes, 2012). The control level is particularly relevant since it includes *goal setting*, which is the decision about the activity in which the writer should engage at a particular point in time (whether making a plan, generating text, or revising the already produced text). This goal setting determines the procedure by which a text is created, as well as the characteristics of the final text (Hayes, 2012; Leijten et al., 2014). As argued in Van den Bergh et al. (2016, p. 58), “the moment at which writers implement a certain activity is critical”. With this study, we contribute to this cognitive theoretical model of writing by showing that goal setting also determines the way in which planning, formulating, and revising alternate and become dominant in different phases of the writing process. Specifically when simplifying texts, expert writers might show greater meta-awareness of how these sub-processes intertwine and temporarily overrule each other with the goal of producing a readable text, while more novice or less proficient L2 writers might need specific training to foster such awareness (Lee and Mak, 2018).

From a methodological perspective, this study is one of the first to show how keystroke logging data can be used for both descriptive and evaluative purposes. For instance, as far as descriptive purposes is concerned, we identified phases of writing based on their multiple and dominant internal dynamics, rather than on the traditional tripartite classification of planning-formulating-reviewing content, that fails to take into consideration the recursive, non-linear nature of writing. Our classification methodology stands out for the multiplicity of dynamics taken into consideration and for its relative ease of implementation to different experimental contexts. It also shed light on the importance of looking at the writing process as a combination of sub-processes, rather than considering each writing task as a single, monolithic activity. We are currently testing the applicability of our criteria to different writing tasks (specifically, to the production of argumentative and persuasive texts in L1), with positive results. Moreover, from a methodological and evaluative point of view, we also showed that the combination of data from keystroke logging and automated text analysis tools can provide important insights into the links between the process and the product of text simplification. A previous study by Medimorec and Risko (2017) applied a combination of the same methods to the analysis of writing of narrative and argumentative texts.

From a pedagogical perspective, this study showed that even limited training sessions on text simplification can assist L2 writers in increasing the readability of texts at a local level (especially, at the sentence level). However, leading students to address macro-level text features (such as, cohesion) and to modify the way in which they manage their text simplification/writing process—in terms of pauses and revisions dynamics across phases—might require longer and possibly more elaborate interventions (Graham and Harris, 2014). Furthermore, as this study provided evidence that more frequent and more prolonged pausing can be beneficial for text quality (especially, readability), it might be important to foster students' understanding of the impact of planning in different phases of the writing process, in line with what is also argued in Xu and Qi (2017). The results on the decrease in readability at different text levels associated with longer and/or more frequent pauses that were “far away” also highlight the need to develop students' awareness of their pausing patterns and to train them to use pauses strategically and recursively (e.g., focusing on paragraph structure after addressing spelling issues). The effectiveness of such process-oriented pedagogy has recently begun to be observed in the classroom (Vandermeulen et al., 2020). On a broader societal level, results from this study raise awareness about the complexity involved in making texts easier to read, and can bring about a new appreciation for the role of linguists/plain language experts.

### Limitations and future research

This study has some limitations that should be addressed in future research. First of all, the limited and quite homogenous group of participants makes it difficult for our findings to be fully generalized to other populations (e.g., younger writers with English as L1). Furthermore, our text simplification task focused on one specific genre, thus limiting the extent to which our results can be generalized to the simplification of other genres. Additionally, while keystroke logging data provide objective measures of observable writing dynamics, they might not fully capture the reasons behind specific writing patterns. Combining keystroke logging with think-aloud protocols can shed more light on the rationale behind writers' use of specific strategies. The criteria that we adopted to identify two writing phases should also be tested and validated further with other genres and tasks. It might also be interesting to develop algorithms that automatically divide writing processes into phases based on a combination of keystroke logging data.

Our text simplification training was quite limited in time and scope. Future research should test the impact of more prolonged training on the development of expertise about text simplification, for example in longitudinal studies. The fact that the control group was able to increase argument overlap (one of the indices of cohesion) also highlights the need to investigate the impact of topic-related training on text simplification. Finally, it might be interesting to check the agreement between automated scores of readability and human evaluations of reading comprehension, which are affected by factors such as prior topic knowledge, years of education, and motivation.

## Data availability statement

The datasets analyzed for this study can be found in an online, open access repository (Zenodo): https://doi.org/10.5281/zenodo.6720290.

## Ethics statement

The studies involving human participants were reviewed and approved by Ethics Committee for the Social Sciences and Humanities at the University of Antwerp (Reference SHW_20_87). The patients/participants provided their written informed consent to participate in this study.

## Author contributions

AR and LV contributed to the design and set-up of this study. AR carried out the data analysis. LV reviewed the procedure and the results. Both authors contributed to the article and approved the submitted version.

## Funding

This study has received funding from the European Union's Horizon 2020 research and innovation programme under the Marie Sklodowska-Curie Grant Agreement No. 888918.

## Conflict of interest

The authors declare that the research was conducted in the absence of any commercial or financial relationships that could be construed as a potential conflict of interest.

## Publisher's note

All claims expressed in this article are solely those of the authors and do not necessarily represent those of their affiliated organizations, or those of the publisher, the editors and the reviewers. Any product that may be evaluated in this article, or claim that may be made by its manufacturer, is not guaranteed or endorsed by the publisher.

## Footnotes

^1^The study described in this article draws on the same dataset analyzed in Rossetti and Van Waes [under review (b)], but it expands the analysis beyond cognitive effort to include writing phases and text quality (specifically, readability).

^2^The training received by the experimental group is available at the following link: https://hosting.uantwerpen.be/calliope/plantra/. The training received by the control group is available at the following link: https://hosting.uantwerpen.be/calliope/plantra-control-training/.

## References

[B1] Alamargot D. Plane S. Lambert E. Chesnet D. (2010). Using eye and pen movements to trace the development of writing expertise: case studies of a 7^th^, 9^th^ and 12^th^ grader, graduate student, and professional writer. Read. Writ. 23, 853–888. 10.1007/s11145-009-9191-9

[B2] Baaijen V. Galbraith D. (2018). Discovery through writing: relationships with writing processes and text quality. Cogn. Instr. 36, 199–223. 10.1080/07370008.2018.1456431

[B3] Baaijen V. Galbraith D. De Glopper K. (2012). Keystroke analysis: Reflections on procedures and measures. Writt. Commun. 29, 246–277. 10.1177/0741088312451108

[B4] Baba K. Nitta R. (2010). “Dynamic effects of task type practice on the Japanese EFL university student's writing: text analysis with Coh-Metrix,” in Proceedings of the Twenty-Third International Florida Artificial Intelligence Research Society Conference (FLAIRS 2010), 217–222.

[B5] Barkaoui K. (2007). Revision in second language writing: What teachers need to know. TESL Canada J. 25, 81–92. 10.18806/tesl.v25i1.109

[B6] Barkaoui K. (2016). What and when second-language learners revise when responding to timed writing tasks on the computer: the roles of task type, second language proficiency, and keyboarding skills. Modern Lang. J. 100, 320–340. 10.1111/modl.12316

[B7] Beauvais C. Olive T. Passerault J.-M. (2011). Why are some texts good and others not? relationship between text quality and management of the writing processes. J. Educat. Psychol. 103, 415–428. 10.1037/a0022545

[B8] Beauvais L. Favart M. Passerault J.-M. Beauvais C. (2014). Temporal management of the writing process: effects of genre and organizing constraints in grades 5, 7, and 9. Writt. Commun. 31, 251–279. 10.1177/0741088314536361

[B9] Bereiter C. Scardamalia M. (1987). The Psychology of Written Composition. Hillsdale, NJ: Lawrence Erlbaum Associates.

[B10] Chen J. Zhang L. (2019). Assessing student-writers' self-efficacy beliefs about text revision in EFL writing. Assess. Writ. 40, 27–41. 10.1016/j.asw.2019.03.002

[B11] Chen J. Zhang L. Parr J. (2022). Improving EFL students' text revision with the self-regulated strategy development (SRSD) model. Metacogn. Learn. 17, 191–211. 10.1007/s11409-021-09280-w

[B12] Cho K. MacArthur C. (2010). Student revision with peer and expert reviewing. Learn. Instruct. 20, 328–338. 10.1016/j.learninstruc.2009.08.006

[B13] Choi I. Deane P. (2021). Evaluating writing process features in an adult EFL writing assessment context: a keystroke logging study. Lang. Assess. Q. 18, 107–132. 10.1080/15434303.2020.1804913

[B14] Crossley S. (2020). Linguistic features in writing quality and development: an overview. J. Writ. Res. 11, 415–443. 10.17239/jowr-2020.11.03.01

[B15] Crossley S. Allen D. McNamara D. (2011). Text readability and intuitive simplification: a comparison of readability formulas. Read. Foreign Lang. 23, 84–101. 10.125/66657

[B16] Crossley S. Allen D. McNamara D. (2012). Text simplification and comprehensible input: a case for an intuitive approach. Lang. Teach. Res. 16, 89–108. 10.1177/1362168811423456

[B17] Crossley S. Kyle K. McNamara D. (2016). The tool for the automatic analysis of text cohesion (TAACO): automatic assessment of local, global, and text cohesion. Behav. Res. Methods 48, 1227–1237. 10.3758/s13428-015-0651-7 26416138

[B18] Crossley S. McNamara D. (2010). “Cohesion, coherence, and expert evaluations of writing proficiency,” in Proceedings of the Annual Meeting of the Cognitive Science Society, 984–989.

[B19] Crossley S. McNamara D. (2011). “Text coherence and judgments of essay quality: models of quality and coherence,” in Proceedings of the Annual Meeting of the Cognitive Science Society, 1236–1241.

[B20] Crossley S. Roscoe R. McNamara D. (2013). “Using automatic scoring models to detect changes in student writing in an intelligent tutoring system,” in Proceedings of the Twenty-Sixth International Florida Artificial Intelligence Research Society Conference, 208–213.

[B21] Crossley S. Skalicky S. Dascalu M. McNamara D. Kyle K. (2017). Predicting text comprehension, processing, and familiarity in adult readers: new approaches to readability formulas. Discour. Processes 54, 340–359. 10.1080/0163853X.2017.1296264

[B22] De Larios J. Manchón R. Murphy L. (2006). Generating text in native and foreign language writing: a temporal analysis of problem solving formulation processes. Mod. Lang. J. 90, 100–114. 10.1111/j.1540-4781.2006.00387.x

[B23] Dowell N. Graesser A. Cai Z. (2016). Language and discourse analysis with Coh-Metrix: applications from educational material to learning environments at scale. J. Learn. Anal. 3, 72–95. 10.18608/jla.2016.33.5

[B24] Eklundh K. (1994). Linear and nonlinear strategies in computer-based writing. Comput. Composit. 11, 203–216. 10.1016/8755-4615(94)90013-2

[B25] Faigley L. Witte S. (1981). Analyzing revision. Coll. Composit. Commun. 32, 400–414. 10.2307/356602

[B26] Flower L. Hayes J. (1981). A cognitive process theory of writing. Coll. Compos. Commun. 32, 365–387. 10.2307/356600

[B27] Graesser A. McNamara D. Cai Z. Conley M. Li H. Pennebaker J. (2014). Coh-Metrix measures text characteristics at multiple levels of language and discourse. Element. School J. 115, 210–229. 10.1086/678293

[B28] Graesser A. McNamara D. Kulikowich J. (2011). Coh-Metrix: Providing multilevel analyses of text characteristics. Educ. Res. 40, 223–234. 10.3102/0013189x11413260

[B29] Graham S. (2018). A revised writer (s)-within-community model of writing. Educat. Psychol. 53, 258–279. 10.1080/00461520.2018.1481406

[B30] Graham S. Harris K. (2014). Conducting high quality writing intervention research: twelve recommendations. J. Writ. Res. 6, 89–123. 10.17239/jowr-2014.06.02.1

[B31] Guo L. Crossley S. McNamara D. (2013). Predicting human judgments of essay quality in both integrated and independent second language writing samples: a comparison study. Assess. Writ. 18, 218–238. 10.1016/j.asw.2013.05.002

[B32] Hayes J. (2004). “What triggers revision?,” in Revision Cognitive and Instructional Processes, eds. L. Allal, L. Chanquoy, P. Largy (New York: Springer Science+Business Media), 9-20.

[B33] Hayes J. (2012). Modeling and remodeling writing. Writt. Commun. 29, 369–388. 10.1177/0741088312451260

[B34] Hayes J. Flower L. Schriver K. Stratman J. Carey L. (1987). “Cognitive processes in revision,” in Advances in Applied Psycholinguistics: Reading, Writing, and Language Learning, ed. S. Rosenberg (Cambridge: Cambridge University Press), 176–240.

[B35] Jackson G. Allen L. McNamara D. (2016). “Common core TERA: Text ease and readability assessor,” in Adaptive Educational Technologies for Literacy Instruction, eds. S. Crossley, and D. McNamara (New York: Routledge), 49–68.

[B36] Kellogg R. (2008). Training writing skills: a cognitive developmental perspective. J. Writ. Res. 1, 1–26. 10.17239/jowr-2008.01.01.1

[B37] Kellogg R. Whiteford A. (2012). “The development of writing expertise,” in Writing: A Mosaic of New Perspectives, eds. E. Grigorenko, E. Mambrino, D. Preiss (New York: Psychology Press, Taylor and Francis Group), 109–124.

[B38] Kuteeva M. (2011). Wikis and academic writing: changing the writer–reader relationship. Engl. Specific Purp. 30, 44–57. 10.1016/j.esp.2010.04.007

[B39] Lee I. Mak P. (2018). Metacognition and metacognitive instruction in second language writing classrooms. Tesol Q. 52, 1085–1097. 10.1002/tesq.436

[B40] Leijten M. Van Horenbeeck E. Van Waes L. (2019a). “Analysing keystroke logging data from a linguistic perspective,” in Observing Writing, eds. E. Lindgren, and K. Sullivan (Leiden: Brill), 71–95.

[B41] Leijten M. Van Waes L. (2013). Keystroke logging in writing research: Using Inputlog to analyze and visualize writing processes. Writt. Commun. 30, 358–392. 10.1177/0741088313491692

[B42] Leijten M. Van Waes L. (2020). Designing keystroke logging research in writing studies. Chinese J. Sec. Lang. Writ. 1, 18–39. Available online at: https://repository.uantwerpen.be/desktop/irua

[B43] Leijten M. Van Waes L. Schrijver I. Bernolet S. Vangehuchten L. (2019b). Mapping master's students' use of external sources in source-based writing in L1 and L2. Stud. Sec. Lang. Acquisit. 41, 555–582. 10.1017/s0272263119000251

[B44] Leijten M. Van Waes L. Schriver K. Hayes J. (2014). Writing in the workplace: Constructing documents using multiple digital sources. J. Writ. Res. 5, 285–337. 10.17239/jowr-2014.05.03.3

[B45] Levy C. Ransdell S. (1995). Is writing as difficult as it seems? Memory Cogn. 23, 767–779. 10.3758/bf03200928 8538448

[B46] Li M. (2012). Use of wikis in second/foreign language classes: a literature review. CALL-EJ 13, 17–35. Available online at: https://mds.marshall.edu/cgi/viewcontent.cgi?article=1024&context=english_faculty

[B47] Lindgren E. Leijten M. Van Waes L. (2011). Adapting to the reader during writing. Written Lang. Liter. 14, 188–223. 10.1075/wll.14.2.02lin

[B48] López P. Torrance M. Rijlaarsdam G. Fidalgo R. (2017). Effects of direct instruction and strategy modeling on upper-primary students' writing development. Front. Psychol. 1054, 1–10. 10.3389/fpsyg.2017.01054 28713299 PMC5491600

[B49] López P. Torrance M. Rijlaarsdam G. Fidalgo R. (2021). Evaluating effects of different forms of revision instruction in upper-primary students. Read. Writ. 34, 1741–1767. 10.1007/s11145-021-10156-3

[B50] McCarthy P. Lehenbauer B. Hall C. Duran N. Fujiwara Y. McNamara D. (2007). A Coh-Metrix analysis of discourse variation in the texts of Japanese, American, and British Scientists. Foreign Lang. Specific Purp. 6, 46–77. Available online at: https://bit.ly/3CEWPrB

[B51] McNamara D. Graesser A. McCarthy P. Cai Z. (2014). Automated Evaluation of Text and Discourse with Coh-Metrix. New York, NY: Cambridge University Press.

[B52] McNamara D. Louwerse M. McCarthy P. Graesser A. (2010). Coh-Metrix: capturing linguistic features of cohesion. Discourse Process. 47, 292–330. 10.1080/01638530902959943

[B53] Medimorec S. Risko E. (2017). Pauses in written composition: on the importance of where writers pause. Read. Writ. 30, 1267–1285. 10.1007/s11145-017-9723-7

[B54] Myhill D. Jones S. (2007). More than just error correction: students' perspectives on their revision processes during writing. Writt. Commun. 24, 323–343. 10.1177/0741088307305976

[B55] Ozuru Y. Dempsey K. McNamara D. (2009). Prior knowledge, reading skill, and text cohesion in the comprehension of science texts. Learn. Instr. 19, 228–242. 10.1016/j.learninstruc.2008.04.003

[B56] Perrin D. Wildi M. (2008). “Cumulated deviation of a linear trend: Statistical modeling of writing processes,” in Proceedings of the Eleventh International Conference of the EARLI Special Interest Group on Writing (SIG Writing 2008).

[B57] Piolat A. Roussey J. Olive T. Amada M. (2004). “Processing time and cognitive effort in revision: effects of error type and of working memory capacity” in Revision Cognitive and Instructional Processes, eds. L. Allal, L. Chanquoy, P. Largy (New York: Springer Science+Business Media), 21–38.

[B58] Révész A. Michel M. Lee M. (2019). Exploring second language writers' pausing and revision behaviors: a mixed-methods study. Stud. Sec. Lang. Acquisit. 41, 605–631. 10.1017/s027226311900024x

[B59] Rijlaarsdam G. Braaksma M. Couzijn M. Janssen T. Kieft M. Raedts M.. (2009). “The role of readers in writing development: Writing students bringing their texts to the test,” in The SAGE Handbook of Writing Development, eds. R. Beard, D. Myhill, J. Riley, M. Nystrand (Los Angeles: SAGE), 436–452.

[B60] Rijlaarsdam G. Braaksma M. Couzijn M. Janssens T. Kieft M. Raedts M.. (2008). Observation of peers in learning to write, practice and research. J. Writ. Res. 1, 53–83. 10.4135/9780857021069.n31

[B61] Rønneberg V. Torrance M. Uppstad P. Johansson C. (2022). The process-disruption hypothesis: how spelling and typing skill affects written composition process and product. Psychologic. Res. 2, 1–17. 10.1007/s00426-021-01625-z 34997328 PMC9470714

[B62] Rossetti A. Van Waes L. (2022). Accessible communication of corporate social responsibility: development and preliminary evaluation of an online module. Bus. Profession. Commun. Q. 85, 52–79. 10.1177/23294906221074324

[B63] Sato K. Matsushima K. (2006). Effects of audience awareness on procedural text writing. Psychologi. Rep. 99, 51–73. 10.2466/pr0.99.1.51-73 17037450

[B64] Schaeffer M. Nitzke J. Tardel A. Oster K. Gutermuth S. Hansen-Schirra S. (2019). Eye-tracking revision processes of translation students and professional translators. Perspectives 27, 589–603. 10.1080/0907676x.2019.1597138

[B65] Schriver K. (1992). Teaching writers to anticipate readers' needs: a classroom-evaluated pedagogy. Writt. Commun. 9, 179–208. 10.1177/0741088392009002001

[B66] Schriver K. (2012). “What we know about expertise in professional communication,” in Past, Present, and Future Contributions of Cognitive Writing Research to Cognitive Psychology, ed. V. Berninger (New York: Psychology Press), 275–312.

[B67] Shermis M. Burstein J. (2013). Handbook of Automated Essay Evaluation: Current Applications and New Directions. New York, NY: Routledge.

[B68] Smeuninx N. De Clerck B. Aerts W. (2020). Measuring the readability of sustainability reports: a corpus-based analysis through standard formulae and NLP. Int. J. Bus. Commun. 57, 52–85. 10.1177/2329488416675456

[B69] Sommers N. (1980). Revision strategies of student writers and experienced adult writers. Coll. Composit. Commun. 31, 378–388. 10.2307/356588

[B70] Traga Philippakos Z. MacArthur C. Munsell S. (2018). College student writers' use and modification of planning and evaluation strategies after a semester of instruction. J. Adolesc. Adult Liter. 62, 301–310. 10.1002/jaal.897

[B71] Tuzi F. (2004). The impact of e-feedback on the revisions of L2 writers in an academic writing course. Computers and Composition 21, 217–235. 10.1016/j.compcom.2004.02.003

[B72] Valenzuela Á. Castillo R. (2022). The effect of communicative purpose and reading medium on pauses during different phases of the textualization process. Read. Writ. 22, 1–28. 10.1007/s11145-022-10309-y

[B73] Van den Bergh H. Rijlaarsdam G. (1996). “The dynamics of composing: Modeling writing process data,” in The Science of Writing: Theories, Methods, Individual Differences, and Applications, eds. C. Levy, and S. Ransdell (Mahwah, New Jersey: Erlbaum), 207–232.

[B74] Van den Bergh H. Rijlaarsdam G. (2001). Changes in cognitive activities during the writing process and relationships with text quality. Educat. Psychol. 21, 373–385. 10.1080/01443410120090777

[B75] Van den Bergh H. Rijlaarsdam G. Van Steendam E. (2016). “Writing process theory: a functional dynamic approach,” in Handbook of Writing Research, eds. C. MacArthur, S. Graham, J. Fitzgerald (New York, NY: The Guilford Press), 57–71. 10.1163/9789004248489

[B76] Van Waes L. Leijten M. (2015). Fluency in writing: a multidimensional perspective on writing fluency applied to L1 and L2. Comput. Composit. 38, 79–95. 10.1016/j.compcom.2015.09.012

[B77] Van Waes L. Leijten M. Van Weijen D. (2009). Keystroke logging in writing research: observing writing processes with Inputlog. German Foreign Lang. 2, 41–64. Available online at: https://repository.uantwerpen.be/desktop/irua

[B78] Van Waes L. Schellens P. (2003). Writing profiles: the effect of the writing mode on pausing and revision patterns of experienced writers. J. Pragmatic. 35, 829–853. 10.1016/s0378-2166(02)00121-2

[B79] Van Waes L. Van Weijen D. Leijten M. (2014). Learning to write in an online writing center: the effect of learning styles on the writing process. Comput. Educ. 73, 60–71. 10.1016/j.compedu.2013.12.009

[B80] Van Weijen D. (2009). Writing processes, text quality, and task effects: Empirical studies in first and second language writing [PhD thesis]. Utrecht, The Netherlands: Universiteit Utrecht.

[B81] Vandermeulen N. Leijten M. Van Waes L. (2020). Reporting writing process feedback in the classroom using keystroke logging data to reflect on writing processes. J. Writ. Res. 12, 109–139. 10.17239/jowr-2020.12.01.05

[B82] Williamson M. Pence P. (1989). "Word processing and student writers,” in Computer Writing Environments: Theory, Research, and Design, eds. B. Britton, and S. Glynn (Mahwah, NJ: Lawrence Erlbaum Associates Inc.), 93–127.

[B83] Xu C. Ding Y. (2014). An exploratory study of pauses in computer-assisted EFL writing. Lang. Learn. Technol. 18, 80–96. 10.125/44385

[B84] Xu C. Qi Y. (2017). Analyzing pauses in computer-assisted EFL writing—a computer-keystroke-log perspective. J. Educat. Technol. Soc. 20, 24–34. Available online at: https://www.jstor.org/stable/pdf/26229202.pdf

[B85] Xu C. Xia J. (2021). Scaffolding process knowledge in L2 writing development: Insights from computer keystroke log and process graph. Comput. Assisted Lang. Learn. 34, 583–608. 10.1080/09588221.2019.1632901

